# Current Status and Prospects of Health-Related Sensing Technology in Wearable Devices

**DOI:** 10.1155/2019/3924508

**Published:** 2019-06-16

**Authors:** Jaegeol Cho

**Affiliations:** Department of Medical and Mechatronics Engineering, Soonchunhyang University, Asan, Chungnam 31538, Republic of Korea

## Abstract

The healthcare-related functions of wearable devices are very useful for continuous monitoring of biological information. Wearable devices equipped with communication function can be used for additional healthcare services. Among the wearable devices, the wristband type is most suitable for acquiring biological signals, and the wear preference of the user is high, so it is highly likely to be used more in the future. In this paper, the health-related functions of wristband were investigated and the technical limitations and prospects were also reviewed. Most current wristband-type devices are equipped with the combination of accelerometer, optical sensor, and electrodes for their health functions, and continuously measured data are expanding the possibility of discovering new medical meanings. The blood pressure measurement function without using cuff is the most useful and expected function among the health-related functions expected to be mounted on the wrist wearable device, in spite of its technical limits and difficulties.

## 1. Introduction

It is a common human desire to live long and healthy regardless of age or area. Recently, as the development of genetic analysis technology has enabled us to recognize and prevent the risk of genetic diseases in advance, this hope has become more realistic. With the development of continuous medical technology and new drug development, diseases that were previously regarded as incurable diseases can now be seen as chronic diseases that can be managed through continuous management. In addition, as the ongoing research and achievement of stem cell technology continues, there is growing hope that human tissue can be regenerated, and thus the possibility of extending the life span of human beings, which is considered to be about the limit of around 100 years, is getting stronger.

So, if we cope with these diseases properly, can anyone live long and healthy? The answer should be considered in two respects. If the best unlimited healthcare services are offered to an individual, the chances of a long and healthy life are high, but considering the high costs involved, it is difficult to imagine that the social medical system will be established where the best medical services are provided to every individual. In other words, if it is not possible to provide unlimited medical services to all members of society, it is imperative to have a sustainable healthcare system that will keep the population as healthy as possible at a minimum cost, in parallel with the development of effective medical technology for treating the disease. Especially as aging has accelerated, the increase in the number of chronic illnesses and consequent increase in the cost of disease management, the increase in waiting time due to lack of medical personnel, and the decrease in actual hours of treatment further strengthen the demand for a new medical system.

In this respect, according to a previous study [1], health is influenced by genetic causes, social environment, environmental factors, behavioral patterns, and medical care. Among these five factors, the most common cause of premature death (death except natural causes caused by aging) is wrong behavioral patterns, accounting for 40% of the total, and medical care contributes only 10%. Therefore, in terms of population health, even if a large amount of money is provided to provide the best medical service, the effect of improvement is considerably weak, and it is the most effective prescription for the members of society to maintain a clean environment with healthy lifestyle.

Environmental factors can be controlled and quantitatively measured and monitored by national and governmental regulations and efforts; however, the individual's lifestyle and behavior will need to be reviewed to determine what to measure and monitor. In order to measure health-related habits in daily life rather than patients in a hospital, a wearable device capable of measuring a biosignal attached to an individual's body is required. Recently, wrist-type smart devices have been widely used, and the healthcare-related technologies that utilize them are increasing. Therefore, not only wellness services that help individuals to manage their own health but also services for remotely managing chronic diseases using measured data are being tried by governments and insurance companies. An example is the use of a wristband to monitor exercise and heart rate after coaching appropriate levels of exercise in patients treated with heart failure, thereby preventing readmission and reducing medical costs accordingly.

In this review, healthcare-related technologies applied to personal wearable devices have been examined which are widely used in recent years, and the sensing technology and related technical issues required for health management in the future are discussed.

## 2. Methods

In this review, health-related functions included in wearable devices are surveyed and summarized. Technical problems related to the cuffless blood pressure measurement function which is not mounted on wrist wearable devices up to now are discussed.

First, the functions of patient monitoring system in hospitals and biosignal measurement items that can be measured in daily life were compared to predict the technology status and development direction of wearable devices. It was also examined that which of the various wearable devices are suitable for biosignal measurement, and the measurable functions were listed accordingly. The biomedical measurement items using sensors included in a wristband, which is a typical wearable device, are summarized.

Secondly, an overview diagram of blood pressure measurement methods has been made to review the possibility and current status of cuffless blood pressure measurement techniques, and the technical limitations of the pulse wave velocity (PWV) method, which has been widely tried, were investigated.

## 3. Discussion

### 3.1. Healthcare Sensing Technology Applied to Wearable Devices

**Figure 1:**
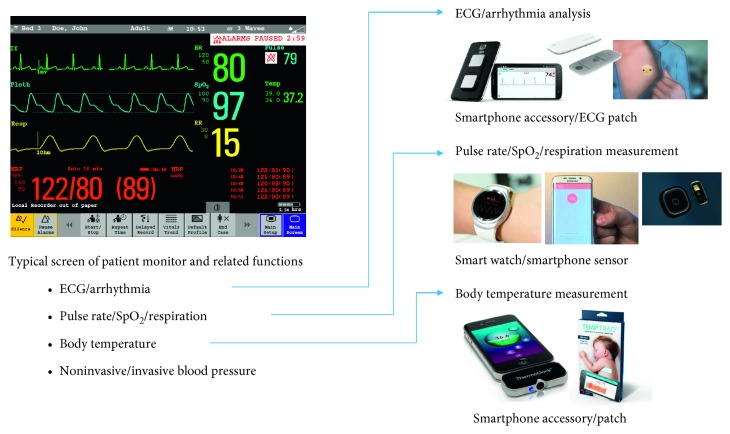
Evolution of patient monitoring system for wearable devices and daily life.

The current development of medical devices shows that it is a big trend to monitor and monitor at any time in home and everyday life, not in a specific place of hospital. In accordance with this trend, medical technology is moving from the field of doctors and specialists to the general public, from the hospital to the home, and also to the mobile environment, and wearable devices and related services will play an increasingly important role. For example, biomedical measurements such as electrocardiograms and oxygen saturation that have been measured using equipment in hospitals can now be easily measured by wearable devices that are small and inexpensive. As a result, new healthcare services using the measurement data can also be introduced. In Figure 1, a typical screen of patient monitoring system used in hospital is shown and its measurement functions are listed up. Recording and analysis of ECG signals and measurements of heart rate, SpO_2_, respiration rate, and body temperature are now all possible with small-size handheld devices or wearable devices as shown in the figure. However, the miniaturization of blood pressure measurement function is still ongoing, which will be discussed later.

**Figure 2:**
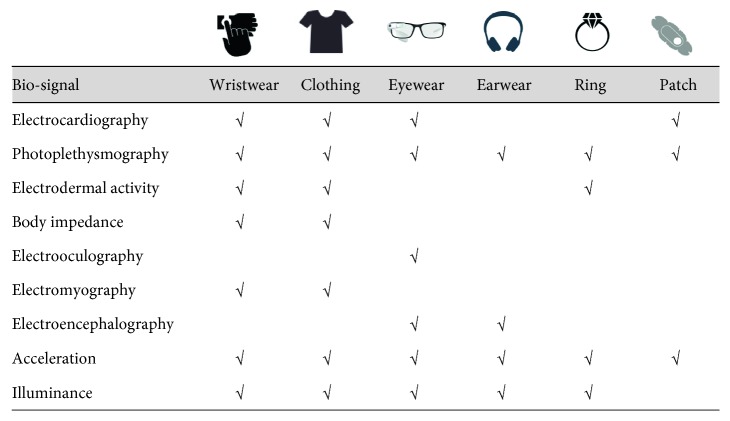
Comparison of biosignal measurement items according to wearable device type and wearing position.

In addition to technological advances, expectations and requirements of consumers who purchase and use wearable devices are also meeting new remote healthcare services. According to a recent report [2], the widespread use of wearable devices is expected to prolong consumers' life span, lower healthcare costs, and reduce obesity issues, and these trends are becoming clearer over time. There are various types of wearable devices currently available, such as wristband-type devices [3–6], glasses [7], shoes [8], patches [9–12], socks [13], clothes [14], earphones [15–17], rings [18], and clips [3]; however, the most suitable form to measure various biosignals and to manage health through it is thought to be the wrist-type device. As shown in Figure 2, the location where the electrocardiogram, photoplethysmography, and electrodermal activity can be easily measured is the wrist, and the experience of wearing a wristwatch is expected to eliminate the feeling of wearing a wearable wristband device. This can be seen in a previous survey [19] of the most preferred locations for wearing wearable devices on the body. The most preferred location was the wrist (65%), and most of the positions replacing the worn accessories such as glasses (55%) and armband (40%) were highly preferred.

**Figure 3:**
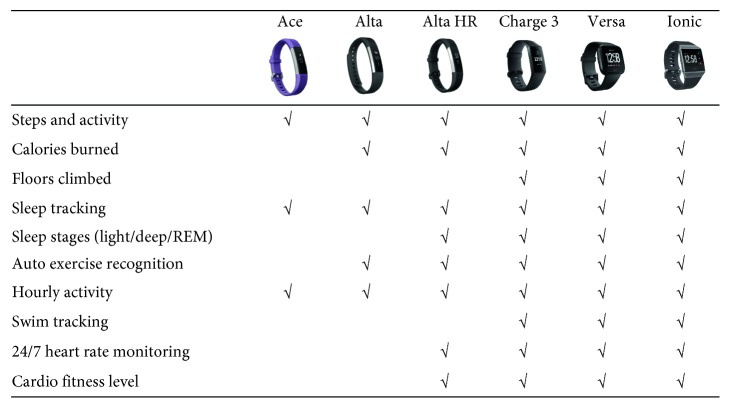
Fitbit's wearable device types and functions (Feb. 2019).

From Fitbit's product portfolio (Figure 3), which is one of the representative companies developing and selling wearable devices, it can be seen that there are various functions by product type and price. In addition, the higher the price, the more various the healthcare-sensing functions included, and the wrist-type device has the most various functions among the company's other products. Representative sensors and related functions applied to wrist wearable devices of major companies such as Fitbit, Apple, Garmin, and Samsung can be roughly classified into three categories as Table 1.

The wristband-type devices currently available are almost all combinations of the above three sensors and functions. In particular, in the case of the optical sensor for measuring the heart rate [20–29], the function of measuring the pulse continuously for 24 hours is spreading using low power architecture, like the case of the accelerometer which is used to measure the movement of the user at all times for 24 hours at very low power. These always-on functions extend the possibility of discovering new medical meanings because the user's biosignals can be continuously measured over several days.

### 3.2. Wearable Blood Pressure Sensing Technology Outlook

Blood pressure is one of the fundamental and important vital signs (pulse, blood pressure, body temperature, respiration) and is closely related to arterial health, which is considered to be an important indicator of health. Therefore, blood pressure is a kind of health indicator that needs to be managed with great importance. In particular, in the case of hypertension, the blood pressure trend for 24 hours is more important than the blood pressure value measured from time to time. Therefore, it is necessary to continuously monitor and manage it. Because hypertension is the most common cause of death among developed and developing countries [30], the need for blood pressure management using wearable devices has long been emphasized, and among the health-related functions, it is expected to be the most useful and expected function to be mounted on the wrist wearable device in the future.

**Figure 4:**
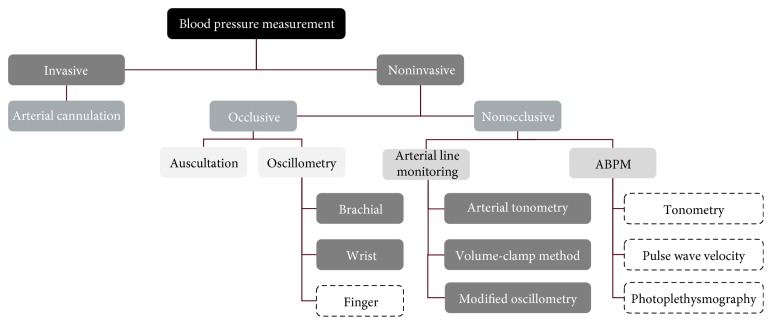
Blood pressure measurement technology classification (the technology in boxes with dashed lines has not been clinically validated).

The methods of blood pressure measurement are classified and summarized in Figure 4. The technologies which have not been clinically proved enough are surrounded with dashed lines. The auscultatory method using a brachial cuff and a stethoscope has been considered as a gold standard of blood pressure measurement [31]; however, the oscillometric blood pressure monitor using cuff is the most widely used method due to convenience and proven accuracy [32]. Since the oscillometric blood pressure monitor uses an upper arm or wrist cuff, it is not suitable for measuring blood pressure from time to time during daily life; nonocclusive techniques which do not occlude the arteries to measure blood pressure are generally considered as the candidates of blood pressure measurement technology for wearable devices. To make blood pressure measurement easy and convenient, Omron Healthcare (Japan) has recently developed a wrist monitor called “HeartGuide,” an oscillometric blood pressure monitor with a relatively small wrist cuff [33]. Although it is necessary to press the finger manually to apply the oscillometric principle, recent studies have shown potential to use smartphones as blood pressure monitors [34, 35]. Among the nonocclusive techniques, tonometry [36–38], pulse wave velocity (PWV) [39–50], and photoplethysmography (PPG) [51, 52] have been extensively studied and developed for ambulatory blood pressure monitoring (ABPM).

**Figure 5:**
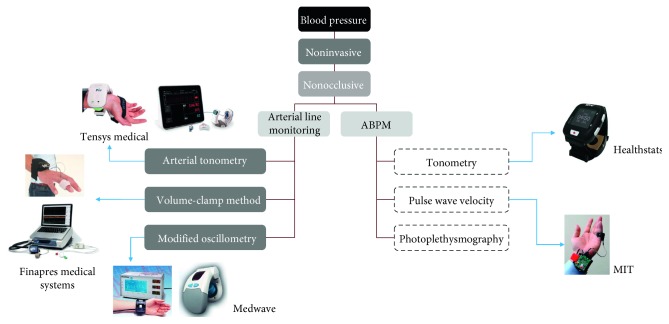
Cuffless blood pressure measurement technologies and products.

Figure 5 shows representative blood pressure measurement devices that do not use a cuff. The T-Line system (Tensys Medical Inc., USA), Finometer (Finapres Medical Systems, Netherlands), and the Vasotrac (Medwave, USA) are typical devices which have been used for noninvasive arterial line monitoring in hospitals. In the T-Line system using radial artery applanation tonometry, the contact pressure between the radial artery and the bone should be optimized and continuously adjusted automatically, and its clinical accuracy has been tested under various conditions [53–55]. The Finometer measures the arterial pressure waveform at the finger using volume-clamp method, also known as the Finapres (FINger Arterial PRESsure) method. The volume of finger artery is measured with optical sensors and is automatically maintained constant with the finger cuff connected to pneumatic control system. The volume-clamp method showed good agreements with intra-arterial monitoring [56] and auscultatory method [57]; however, when measuring blood pressure waveforms under abnormal conditions such as finger oedema or insufficient blood perfusion, the volume-clamp method is inaccurate or even impossible [58, 59]. The Vasotrac device measures the radial blood pressure using pressure sensing module located over the radial artery with a length-adjustable wrist strap. The wrist strap automatically repeats compression and release to record the radial blood pressure waveform and estimate the blood pressure every 12∼15 pulse beats using proprietary algorithms. Previous studies showed that the accuracy of Vasotrac is controversial [60, 61].

There also have been numerous attempts to develop ABPM devices; however, personal blood pressure monitors without cuffs have not yet been proven to be accurate. Although the BPro device (Healthstats, Singapore) showed good accuracy in previous studies [36–38], the tonometry method is highly sensitive to motion and needs precise positioning of the sensor, which makes it difficult to be commonly used in daily life [62]. As is shown in the figure, all the previous studies and products without using cuffs have attempted to measure blood pressure in the wrist or finger for a variety of reasons, such as the location of the artery, easiness for ECG measurement, and so on.

The most popular method among the various methods shown in Figure 5 is the method using pulse wave velocity (PWV), which is a method of estimating the blood pressure using the phenomenon that the pressure inside the artery (that is, blood pressure) changes the elasticity of the artery and changes the time of the pulse wave from the heart to the peripheral artery.

**Figure 6:**
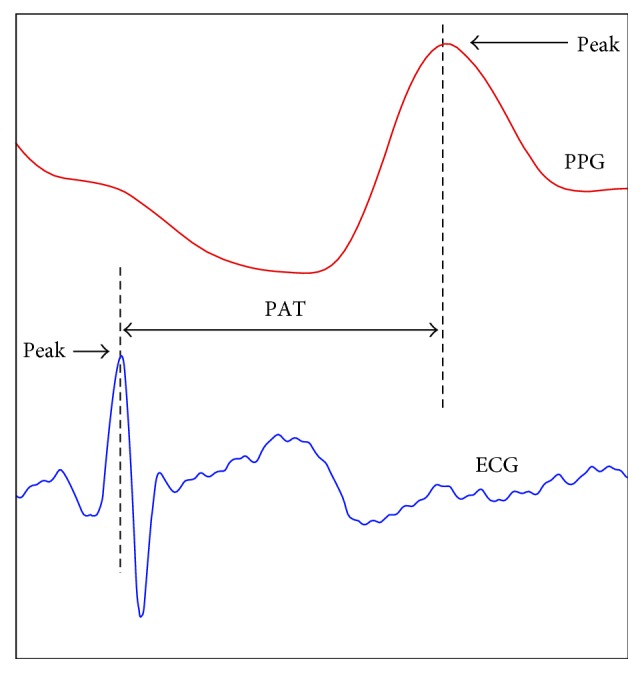
Measurement of pulse arrival time (PAT) using photoplethysmogram (PPG) and electrocardiogram (ECG).

The PWV can be calculated by dividing the artery length with the pulse transit time (PTT) or pulse arrival time (PAT), which can be derived combining the feature points (peak, valley, peak of 1^st^ derivative, etc.) of electrocardiogram (ECG), photoplethysmogram (PPG) [39–46], phonocardiogram (PCG) [47], seismocardiogram (SCG) [48], and ballistocardiogram (BCG) [49, 50]. Sometimes the PTT and PAT are used as the same; however, the strict meaning of the pulse arrival time is the sum of pulse transit time and preejection period (PEP) [63].  Figure 6 shows a typical example of PWV methodology, which uses the peaks of ECG and PPG. The PAT in Figure 6 includes the preejection time due to the use of the ECG. Detailed descriptions of PTT and PAT measurements can be found in previous studies [63].

The difficulties or problems of conventional blood pressure estimation techniques using PWV can be summarized as follows:

1. Because the elasticity of an artery changes not only by blood pressure but also by other factors such as the environment, temperature, and emotion, it is difficult to estimate the accurate blood pressure when the arterial characteristics change with time. In addition, when the pulse arrival time including PEP is used for PWV calculation, the PEP fluctuation is expected to reduce the accuracy of the blood pressure estimation [63–66].
2. Calibration using a conventional sphygmomanometer with cuff is always necessary since the PWV method does not measure the blood pressure values directly.
3. Since systolic and diastolic blood pressures are independent of each other, additional information other than the PWV measurement is needed to obtain the two blood pressure values (two equations are needed for two unknowns to be solved; however, only one PWV can be measured). Therefore, accurate estimation of systolic and diastolic blood pressure requires efforts to find new and effective features besides PWV, as shown in a recent work [50].

In conclusion, the cuffless blood pressure estimation technology using PWV for wearable devices should be designed and developed to overcome such shortcomings.

## 4. Conclusions

An increase in the cost of healthcare due to aging necessitates the establishment of a more effective healthcare system. The healthcare function of the wearable device can be very useful for efficient preventive/postmanagement services in that the user's body information can be monitored, and communication with healthcare professionals is always possible. Among various wearable devices, the wristband is most suitable for acquiring biomedical signals, so it is highly likely to be useful in a new medical system. The sensors of wristband for health-related functions are mainly composed of accelerometer, optical sensor, and electrodes. Using the combinations of these sensors, wristband measures most functions of the patient monitoring system in hospital except blood pressure measurement.

Therefore, the biggest leap in health-related functions of wristband-type devices is expected to be blood pressure measurement technology without cuff, and it is expected to have a ripple effect as the technical difficulty is very high. However, even if a new blood pressure sensing technology is not developed early, the 24-hour continuous biosignal measurement function of the wearable device, which has been developed so far, provides biosignals in daily life in various environments. Unlike patient data measured in hospitals, it will provide new medical implications and possibilities for healthcare services, and these efforts are expected to continue.

## Figures and Tables

**Table 1:** Sensors and their health-related functions in wearable devices.

Sensor	Functions
Accelerometer	Step count/calories/pace and distance/active time Sleep efficiency/sleep time/sleep stages (light, deep, REM) Exercise recognition (walking, running, rowing, swimming, etc.)

Optical sensor	Heart rate (at rest, during exercise) from photoplethysmogram (PPG) 24 h continuous heart rate Respiration Oxygen saturation (SpO_2_) Heart rate variability (HRV) Light intensity (illuminance)

Electrodes	Electrocardiogram (ECG) Bioimpedance analysis (body fat, muscle, etc.) Heart rate (impedance plethysmography) Perspiration
